# Supplementary material for: Recruitment of DNA Repair MRN Complex by Intrinsically Disordered Protein Domain Fused to Cas9 Improves Efficiency of CRISPR-Mediated Genome Editing
Source: Biomolecules. 2019 Oct 8;9(10):584. doi: 10.3390/biom9100584 (PMC6843829; doi:10.3390/biom9100584)
Supplement: Supplementary file 1 [file biomolecules-09-00584-s001.pdf]

Supplementary Material for

Recruitment of DNA repair MRN complex by intrinsically disordered protein domain fused to Cas9 improves efficiency of CRISPR-mediated genome editing

Nina Reuven*, Julia Adler, Karin Broennimann, Nadav Myers and Yosef Shaul*
Dept. of Molecular Genetics, Weizmann Institute of Science, Rehovot, Israel 76100

*Corresponding authors: Yosef Shaul and Nina Reuven
Email: yosef.shaul@weizmann.ac.il and nina.reuven@weizmann.ac.il

This file includes:

Figures S1 to S8
Tables S1-3

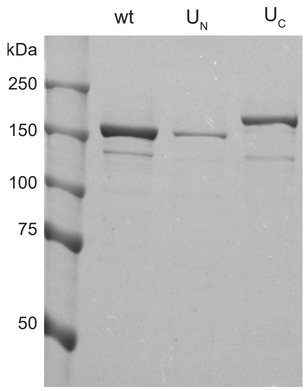

Fig. S1. Purified Recombinant Cas9 proteins 1.2 μg of purified recombinant wt, U_N, and U_C Cas9 proteins were analyzed by SDS-PAGE and stained with GelCode® Blue Stain Reagent. Proteins were purified from IPTG-induced cultures as follows: Cells were harvested by centrifugation, and the pellet was resuspended in 100 ml lysis buffer containing 0.5 M NaCl, 20 mM Tris-HCl, pH 8, 20 mM imidazole supplemented with 0.2mg/ml lysozyme, 20μg/ml DNase, 1mM $MgCl_2$, and protease inhibitor cocktail (Calbiochem set 3), and 20mM imidazole. After lysis by protein disrupter (Constant Systems), the soluble fraction was obtained by centrifugation and purified by immobilized metal ion affinity chromatography (IMAC) using a HiTrap *FF*_5ml cartridge (GE Healthcare) using an FPLC system (ÄKTA GE Healthcare Life Sciences). Cas9 variants were eluted in one step with the binding buffer supplemented with 0.5M Imidazole and injected directly into a size exclusion column (HiLoad_16/60 Superdex 200) equilibrated with 20mM Tris 8, 200mM KCl and 10mM $MgCl_2$. All peak fractions were analyzed for the presence of Cas9 using SDS-PAGE, and the purity was estimated to be >90%. To obtain higher purity, the pooled fractions were diluted with 20mM HEPES pH=7.5, 100mM KCl and applied to a cation exchange column (Tricorn MonoS 10/30 GL, GE Healthcare) equilibrated with the dilution buffer. The protein was eluted with the same buffer using a gradient to 1M KCl. The final pure enzyme was concentrated and supplemented with 50% glycerol and stored at -20°C.

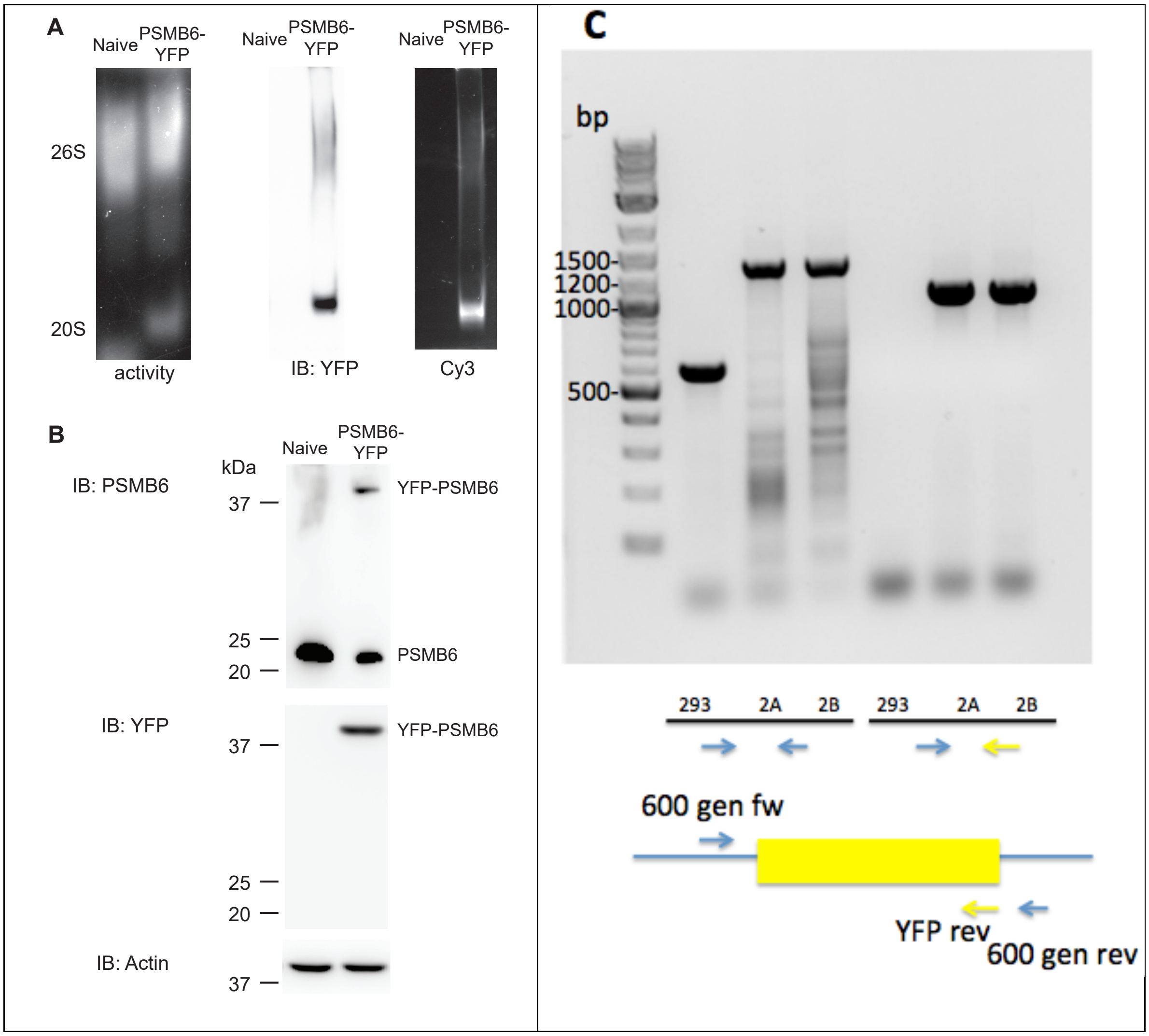

Figure S2. PSMB6-YFP in CRISPR-edited cells is incorporated into active proteasomes. Naive HEK293 and CRISPR-edited PSMB6-YFP-expressing HEK293 cells were analyzed by native gel (A), and SDS-PAGE (B). A) Proteasomal samples were loaded on a nondenaturing 4% polyacrylamide gel. Gel was overlaid with Suc-LLVY-AMC (50 μM) for assessment of proteasomal activity by ImageQuant LAS 4000 (GE) (left panel). The Cy3 filter was used to detect YFP (right panel). Proteins were transferred to nitrocellulose and probed with anti-YFP (middle panel). B) Cells were analyzed by SDS-PAGE and immunoblotting with the indicated antibodies. The samples were not boiled prior to loading, which causes a slight change in migration of the 50kDa PSMB6-YFP protein. **C. PCR analysis of two single cell 293 PSMB6-YFP clones.** Genomic DNA from naive HEK293 cells, and from purified single-cell clones (2A and 2B) were subjected to PCR using the primers indicated. The 600 gen forward and reverse primers generate a 600bp fragment using naive genomic template, and a 1500bp fragment with the YFP insertion. Using the YFP reverse primer and 600 gen forward primer, the expected fragment is 1050 bp. This analysis suggests biallelic insertion of the YFP cassette in the two clones, since the genomic primers no longer generate the 600 bp fragment, and only the 1500 bp fragment is detected clearly.

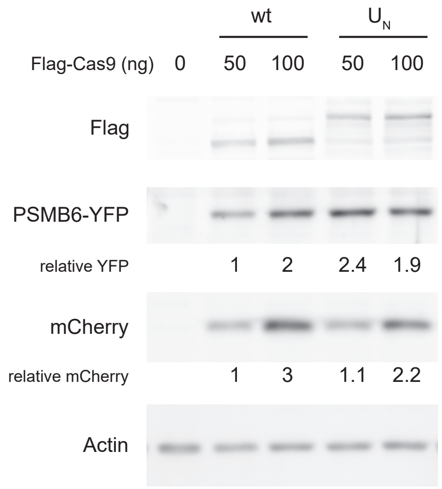

Figure S3. Editing of PSMB6-YFP is improved with MRN-recruiting constructs. HEK293 cells were transfected with the indicated amounts of Cas9/sgRNA encoding plasmids and with donor DNA. Cells were analyzed by SDS-PAGE and immunoblotting with the indicated antibodies. Cas9 is expressed on a Cas9-T2A-mCherry cassette, thus mCherry level reflects Cas9 expression.

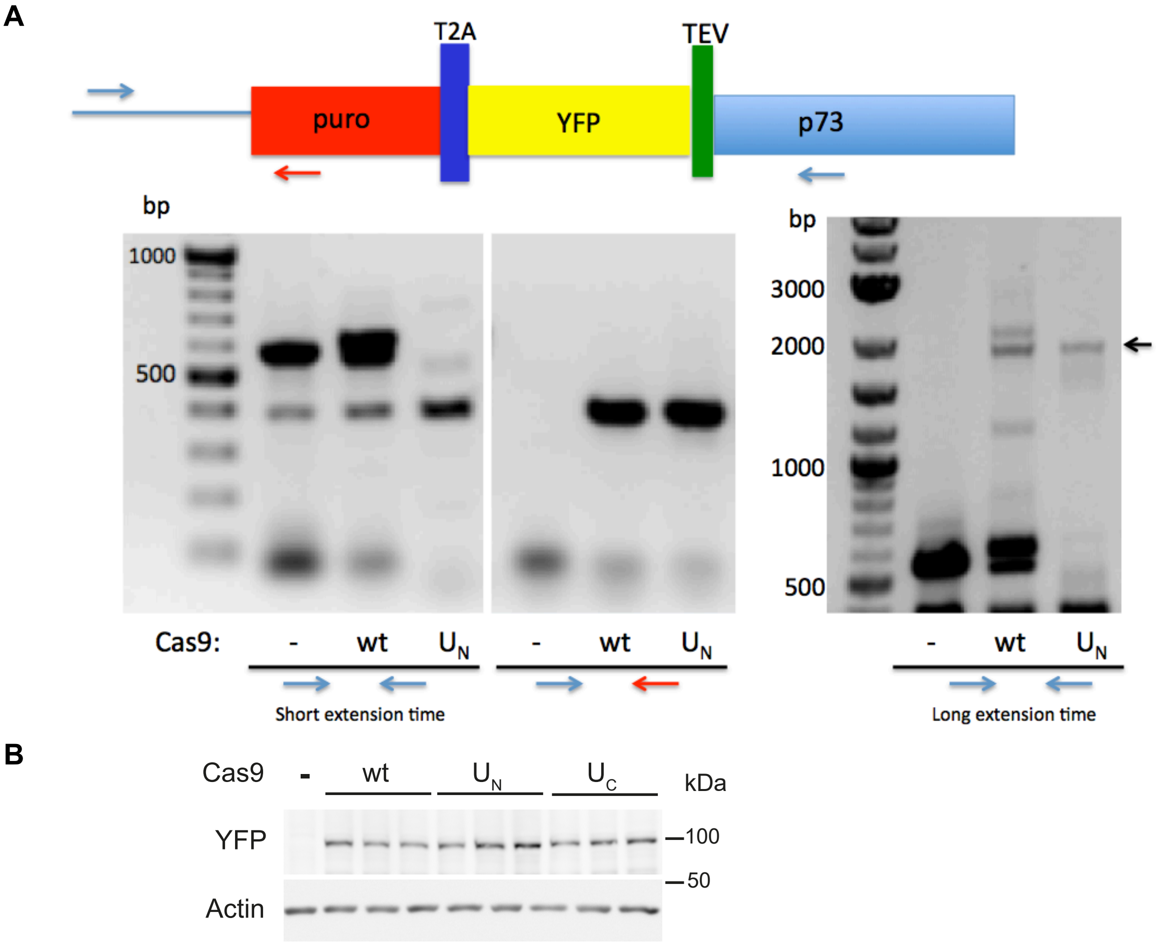

Figure S4. A. PCR analysis of single cell purified HEK293 pac-2A-YFP-p73 clones. PCR was performed on genomic DNA using the primers indicated. The (blue) genomic-specific primers generate a 580bp fragment with the naive HEK293 template, and a 2kb fragment with the proper insertion of the cassette. Using the genomic forward primer and puro (red) reverse primer, the expected fragment is 380bp. **B.Puromycin-selected pools of edited cells express YFP-p73**. Samples of the cells from the experiment presented in Figure 3H were replated two days post-transfection in medium with 0.5μg/ml puromycin. Control cells (transfected without Cas9) were also plated without puromycin. The control cells did not survive puromycin treatment, and thus the control sample is from cells not treated with puromycin. Cells were analyzed by SDS-PAGE and immunoblotting, with the Living Colors antibody used to detect YFP-p73. All of the puromycin-selected cells express YFP-p73.

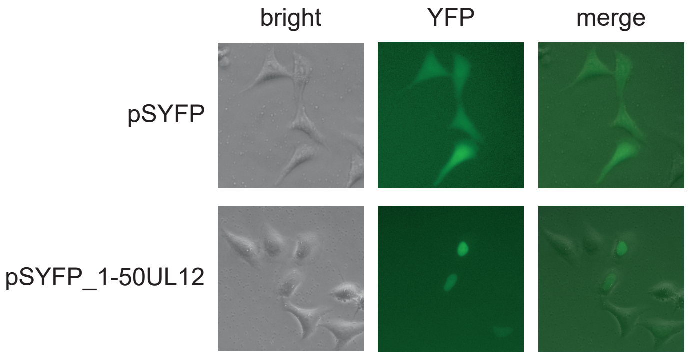

Figure S5. UL12 1-50aa fragment has NLS. HeLa cells were transfected with pSYFP-C1, or with pSYFP-1-50 UL12, with the 1-50aa UL12 fragment fused to the C-terminus of SYFP. Cells were photographed with YFP filter and brightfield. Cells expressing SYFP-1-50UL12 have nuclear-localized YFP, while naive SYFP is dispersed throughout the cell.

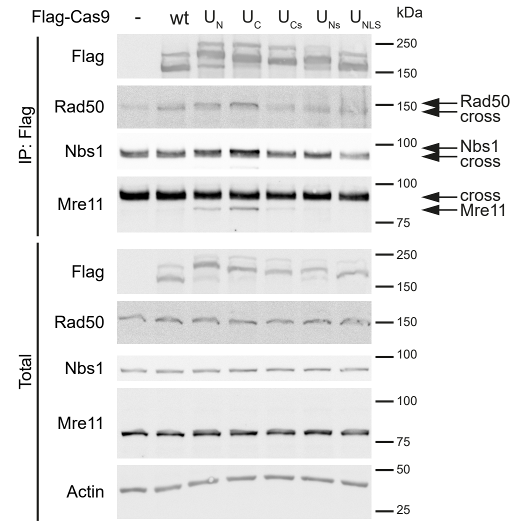

Figure S6. Complete 126 aa domain of UL12 is needed for recruitment of MRN. HEK293 cells were transfected with the indicated constructs and treated as described in Fig. 1C. The U_N and U_C constructs have the 126aa UL12 domain fused to the N- and C-termini of Cas9, respectively. The U_{Ns} and U_{Cs} have the 50-126 aa domain fused to the N- and C- termini of Cas9, and U_{NLS} has the 1-50 aa domain fused to N- terminus of Cas9. In the IP panels for Mre11, Nbs1 and Rad50, the indicated proteins, and cross-reacting bands, are indicated with arrows.

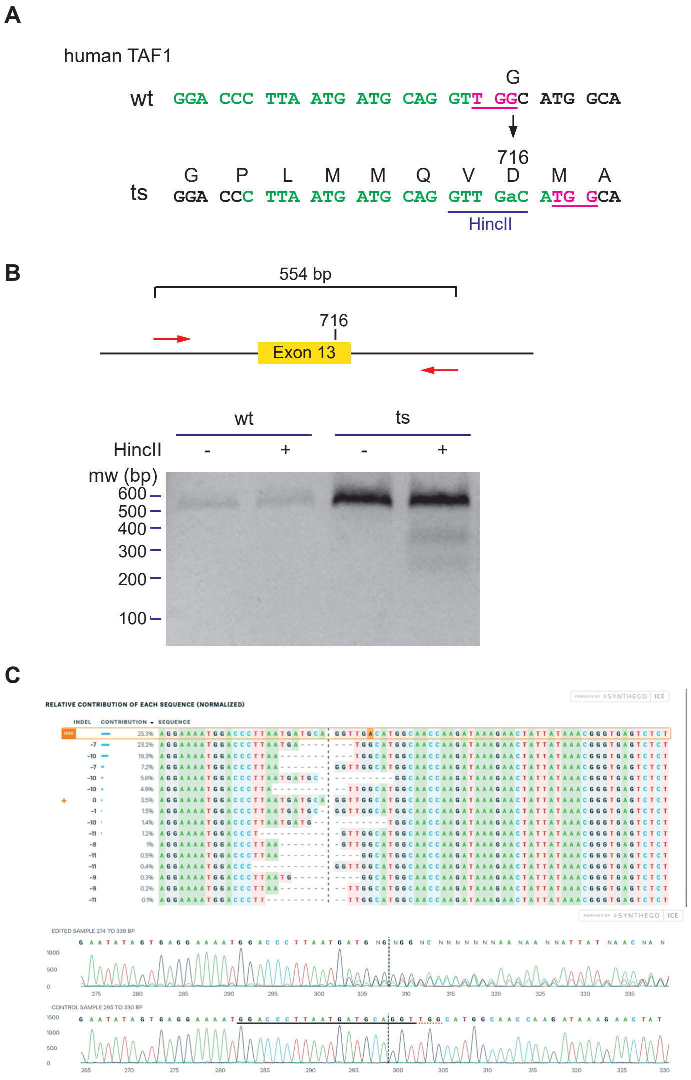

Fig. S7. Editing of HEK293 to produce TAF1 G716D ts cells. A. Targeted locus of human TAF1. Guide sequences for targeting the wt locus, and subsequently the mutated locus, are indicated in green, and the PAM sequences are in pink. The ts mutation creates a HincII site. **B. PCR analysis of representative HEK293 TAF1ts clone.** A 554 bp fragment was amplified from the genomic DNA of a wt (control) and ts clone, and cleaved with HincII. Primers used are listed in Supplementary Table S1. HincII cleavage indicates incorporation of the mutant sequence. **C. Sanger sequencing analysis of representative HEK293 TAF1ts clone.** The 554 bp fragment from the ts clone amplified in (B) was sequenced, and the results were analyzed by using the Synthego ICE beta tool **(**Synthego Performance Analysis, ICE Analysis. 2019. v1.2. Synthego; [accessed 18.10.18]. Beta release). The top three sequences represent the sequences of the three X chromosomes. The other sequence suggestions are likely due to noisiness of the sequencing results. The results indicate one chromosome with the planned G716D mutation (marked HDR), one chromosome with a -7 and one with a -10 deletion. Both deletions cause frame-shifting and early termination (5-6 amino acid addition, then stop codon). The lower panels show the Sanger sequencing results of the edited and control samples. The guide sequence is underlined, the PAM underlined with a dotted red line, and the location of the Cas9 cleavage indicated by the vertical dashed line.

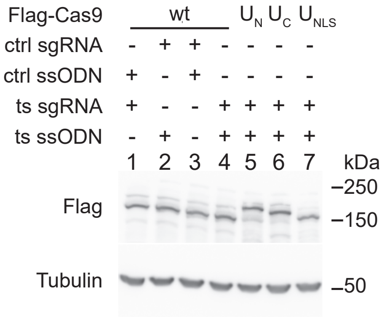

Figure S8. MRN-recruiting constructs of Cas9 show more efficient editing of point mutation - expression levels of Cas9 constructs. SDS-PAGE and immunoblot analysis of samples of cells transfected in the experiment shown in Figure 4C. The results indicate equal expression levels of Flag-Cas9 constructs.

Supplementary Table S1 - Oligonucleotides used in this study

**Cloning UL12 fragments at N-terminus of Cas9:**	
SgrAI_HindIII_UL12_fw (for U_N constructs)	attcga**cgccggtg**aagcttgccaccatggagtccacgggaggcccag
SgrAI_BamHI_UL12_126_re (for 1-126 U_N and 50-126 U_{Ns} constructs)	attcga**cgccggtg**ccggatccagagtcaaggtccggggagtc
SgrAI_SalI_UL12_50_fw (for 50-126 U_{Ns} construct)	attcga**cgccggtg**gtcgacgccaccatgctgcccccccccaccccagacg
SalI_UL12_1_fw (for 1-50 U_{NLS} construct, made by replacing SalI-BamHI fragment in U_{Ns})	attcgacg**gtcgac**gccaccatggagtccacgggaggc
BamHI_UL12_50_re (for 1-50 U_{NLS} construct)	ataggtca**ggatcc**gggacggaaggtggttg
**Cloning UL12 fragments at C-terminus of Cas9:**	
FseI_pX_UL12_fw (for U_C constructs)	gaaaaa**ggccggcc**aggcaaaaaagaaaatggagtccacgggaggc
HindIII_126UL_re (for cloning into Addgene #64324, upstream of P2A-mCherry, for U_C and U_{Cs} constructs)	gccctc**aagctt**agagtcaaggtccggggagtc
FseI_CasUL12_50_fw (for cloning 50-126 U_{Cs} construct)	gaaaaa**ggccggcc**aggcaaaaaagaaactgcccccccccaccccagacg
**Oligos for changing sgRNA cloning site from BbsI to BsaI in pX330 and derivatives:**	
pX330_BsaI_fw	caccg**gagacc**tgtaca**ggtctc**t
pX330_BsaI_rev	aaaca**gagacc**tgtaca**ggtctc**c
**Guide for C-terminus of human PSMB6**	
CR_B6_stop_g2_fw	caccgTAGAATCCCAGGATTCAGGC
CR_B6_stop_g2_re	aaacGCCTGAATCCTGGGATTCTAc
**ssODN template for human PSMB6-Flag**	
GCAAGTACTTTTGGGAGACCAGATACCCAAATTCGCCGTTGCCACTTTACCACCC GCCaagcttGACTACAAAGACGATGACGACAAGTGAATCCTGGGATTCTAGTATGCA ATAAGAGATGCCCTGTACTGATGCAAAAT	
**Primers to make human PSMB6-YFP donor template in pBluescript KS- with 1 kb homology arms**	
SalI_b6_frg1_fw	ctcgag**gtcgac**cactattctgccatcctgcaggtcctacatcg
HindIII_b6_frg1_re	ggtggc**aagctt**ggcgggtggtaaagtggcaacggcgaatttggg
HindIII_ATG_Clover/YFP_fw	cccgcc**aagctt**gccaccatggtgagcaagggcgagg
BamHI_Clover/YFP_rev	gattca**ggatcc**agctcgagatctgagtccggacttgtacagctcg
BamHI_b6_Frg2_fw	cgagct**ggatcc**tgaatcctgggattctagtatgcaataagagatg
XbaI_b6_Frg2_re	ggccgc**tctaga**gcagtgagccaagaccaggctactgcactccagc
**Primers for making donor DNA for N-terminal fusion of YFP to human p73 using pBluescript KS- with 1kb homology arms (see above for fw primer used to amplify YFP)**	
XhoI_p73N_frg1_fw	ggtggg**ctcgag**tttccctgtcccctcccc
HindIIIp73N_frg1_re	cttgct**aagctt**catcttccccacgccggcctccgagggcagctc

EcoRI_TEV_YFP_re	ggccat**gaattc**gccctggaagtacaggttctcagctcgagatctgagtccggacttgtacag
EcoRI_p73N_frg2_fw	tacaag**gaattc**atggcccagtccaccgccacctcccctgatgg
BamHI_p73N_frg2_re	cacct**ggatcc**agccatgcctgaatccattcc
Primers for the insertion of cassette (puromycin resistance gene-T2A peptide) upstream of YFP in the p73 targeting vector	
HindIII_puro_fw	aagatg**aagctt**atgaccgagtacaagccc
HindIII_T2A_puro_rev	cttgct**aagctt**tgggccaggattctcctcgacgtcaccgcatgttagcagacttcctctgccctctccactgccggcaccgggcttgcgggtcatg
**Guide for human p73 N-terminus**	
p73N_guide1_fw	caccgCTGGGCCATCTTCCCCACGC
p73N_guide1_re	aaacGCGTGGGGAAGATGGCCCAGc
**Primers flanking the p73 guide site, to amplify 500bp fragment**	
p73N_500bp_fw	ctcttccaaggcgacggctctgagaagctc
p73N_500bp_re	ccagtgagggttgccaagtttagcccaaag
**Primers for checking integration into the genomic p73 locus**	
p73_1200upNterm_fw (this primer is upstream of the left homology arm in the p73 genomic sequence)	cttctatcagctcccgcctgcctggggaag
puro_re	gtcgtcgcgggtggcgaggcgcaccgtggg
**Guide for targeting mutation site in TAFII250 in BHK ts13**	
ts13_guide_fw	caccgATTAATGATGCAAGTTG**a**CA
ts13_guide_re	aaacTGtCAACTTGCATCATTAATc
**ssODN for correcting ts mutation in BHK ts13**	
TTAAGCCCAGACTCACCCGCTTATAGTAGTTTTTATCTTGGTTGCCATGCCAACTTGCATCATTAATGGTCCATTTTCCTCACTGTATTCTGCAAGAAT	
**Guide for targeting human TAF1, aa716**	
TAF1_g1_fw	caccGGACCCTTAATGATGCAGGT
TAF1_g1_re	aaacACCTGCATCATTAAGGGTCC
**ssODN for creating ts mutation (G716D) in human TAF1**	
TCTGAGCAGAGACTCACCCGTTTATAATAGTTCTTTATCTTGGTTGCCATGtCAACCTGCATCATTAAGGGTCCATTTTCCTCACTATATTCTGCAAGAATAA	
**Primers flanking the TAF1 guide site, to amplify 554bp fragment**	
TAF1_hum_gen554_fw	gcagaacccatacatggatatggagg
TAF1_hum_gen554_re	tatggtatatgttcacagattaccag
**Guide targeting the mutant human TAF1**	
humTAF1_tsmut_g2_fw	caccgCTTAATGATGCAGGTTGaCA
humTAF1_tsmut_g2_re	aaacTGtCAACCTGCATCATTAAGc
**ssODN for correcting ts mutation to make wt human TAF1**	
CTGAGCAGAGACTCACCCGTTTATAATAGTTCTTTATCTTGGTTGCCATGCCAACCTGCATCATTAAGGGTCCATTTTCCTCACTATATTCTGCAAGAAT	
**Control guide non-targeting in human**	
BFP_g2_fw	caccgCTGCACGCCGTGGGTCAGGG
BFP_g2_re	aaacCCCTGACCCACGGCGTGCAGc
**Control ssODN**	
ACCGGCAAGCTGCCCGTGCCCTGGCCCACCCTCGTGACCACCCTGACATACGGCG	

TGCAGTGCTTCAGCCGCTACCCCGACCACATGAAGCAGCACGAC	
**Primers for making sgRNA amplicon for in vitro T7 transcription**	
scaffold_univ_SG9_re	AAAAAgcaccgactcgg
T7_B6_g2_fw (for PSMB6_g2)	GATCACTAATACGACTCACTATAGgTA GAATCCCAGGATTCAGGC

Supplementary Table S2. FACS analysis of PSMB6-YFP edited cells.
Raw data of the FACS experiments summarized in Figure 3C. HEK293 cells were transfected with the Cas9 constructs shown and the PSMB6-YFP donor plasmid. The percentages of YFP-positive cells from three independent experiments is shown.

	**Experiment 1**		**Experiment 2**		**Experiment 3**		**Average**	**SEM**
**sample**	**% YFP**	**fold**	**% YFP**	**fold**	**% YFP**	**fold**	**fold**	
wt Cas9	0.5	1	1.5	1	0.7	1	1	
U_N Cas9	1.1	2.2	2.9	1.9	0.8	1.2	1.8	0.30
U_C Cas9	0.9	1.8	2.6	1.7	0.7	1	1.5	0.27

Supplementary Table S3. XTT assay for quantification of puromycin-resistant cells.
Raw XTT readings from experiment described in Figure 3H. Following transfection with the Cas9/sgRNA and donor plasmids, duplicate samples of cells were replated with and without puromycin, with serial dilutions. The XTT assay was used to quantify cell number. Values for the puromycin-resistant cells were normalized for number of cells plated by dividing by the value obtained for those cells grown without puromycin. The XTT values for cells grown in puromycin were in the same range as the XTT values for the 1:10 diluted cells grown without puromycin. This indicates that overall, the editing efficiency was in the range of 10%.

	**blank**	**no Cas9**		**wt**			**U_N**			**U_C**		
+puro	-0.01	-0.01	-0.01	0.17	0.223	0.519	0.905	1.07	0.955	1.314	0.881	0.51
1:10 no puro	0.011	0.242	0.385	0.302	0.38	0.425	0.542	0.495	0.599	0.486	0.388	0.29
+puro/no puro		-0.04	-0.01	0.56	0.59	1.22	1.67	2.16	1.59	2.70	2.27	1.76
**Ave +puro/no puro**		**0**		**0.79**			**1.81**			**2.24**		
SEM				0.22			0.18			0.27		
